# Role of the *PPARGC1A* Gene and Its rs8192678 Polymorphism on Sport Performance, Aerobic Capacity, Muscle Adaptation and Metabolic Diseases: A Narrative Review

**DOI:** 10.3390/genes15121631

**Published:** 2024-12-20

**Authors:** David Varillas-Delgado

**Affiliations:** 1 Exercise and Sport Science, Faculty of Health Sciences, Universidad Francisco de Vitoria, 28223 Pozuelo, Spain; david.varillas@ufv.es; 2 SPORTNOMICS S.L., 28922 Madrid, Spain

**Keywords:** *PPARGC1A* gene, PGC-1α protein, sport performance, muscle adaptation, metabolic diseases

## Abstract

Background/Objectives: The *PPARGC1A* gene, encoding the PGC-1α protein, is a critical regulator of energy metabolism, influencing mitochondrial biogenesis, fatty acid oxidation, and carbohydrate metabolism. This narrative review aims to evaluate the role of the *PPARGC1A* gene, with a specific focus on the c.1444G<A polymorphism (rs8192678), in sports performance, including its impact on aerobic capacity, muscle adaptation, and its potential implications for metabolic health. Methods: A comprehensive literature search was conducted using databases such as PubMed, Scopus, Science Direct, and Web of Science, following PRISMA guidelines. Studies investigating the rs8192678 polymorphism in athletes, its relationship with physical performance, and its broader metabolic effects were included. Data were synthesized qualitatively, and heterogeneity among findings was assessed. The rs8192678 polymorphism influences sports performance differently. Results: the G allele is associated with enhanced mitochondrial efficiency, higher aerobic capacity, and a greater proportion of fatigue-resistant type I muscle fibers, benefiting endurance sports like cycling and triathlon. Conversely, the A allele correlates with reduced mitochondrial biogenesis and oxidative capacity, potentially impairing endurance but showing possible utility in strength-based sports. Furthermore, the A allele is linked to increased risks of metabolic conditions, including type 2 diabetes and obesity. Discrepancies in results highlight the influence of genetic, environmental, and training interactions. Conclusions: the *PPARGC1A* rs8192678 polymorphism plays a significant role in athletic performance and metabolic regulation. While the G allele confers advantages in endurance sports, the A allele presents mixed implications for strength and metabolic health. These findings support the potential for genetic profiling in personalized training and health interventions but emphasize the need for further research to clarify genotype-environment interactions.

## 1. Introduction

The *PPARGC1A* gene (peroxisome proliferator-activated receptor γ coactivator 1-α) is a key regulator of energy metabolism, playing a fundamental role in various biological processes essential for muscular adaptation, especially in response to stimuli such as physical exercise [1]. This gene encodes the PGC-1α protein (peroxisome proliferator-activated receptor γ coactivator 1-α), which serves as a critical transcriptional coactivator in multiple metabolic pathways, with a direct impact on mitochondrial biogenesis [2], oxidation of fatty acids [3,4], metabolism of carbohydrates [5], and thermogenesis [6,7]. These are fundamental processes in cellular biology and physical performance. The relationship between the expression of the *PPARGC1A* gene and the functional PGC-1α protein is crucial for understanding how these metabolic pathways contribute to energy production and utilization efficiency during aerobic and anaerobic exercise conditions, as well as for maintaining cellular energy balance at rest [8,9,10,11,12].

The *PPARGC1A* gene is recognized for its ability to activate the transcription of genes dependent on peroxisome proliferator-activated receptors (PPARs), which are involved in the regulation of lipid and carbohydrate metabolism, as well as in the differentiation and specialization of muscle fibers [13]. This regulation is crucial in improving athletic performance, especially in modalities that require high levels of endurance, such as cycling, triathlon, and other aerobic endurance disciplines [14]. The functions of *PPARGC1A* in energy homeostasis and muscle adaptation to training stimuli have led to intensive study in the field of sports genetics [15,16].

One of the most studied aspects in the athletic performance of the *PPARGC1A* gene is the c.1444G>A (rs8192678) polymorphism. This polymorphism involves a change from glycine to serine at position 482 of the PGC-1α protein [p.G482S (Gly482Ser)], which appears to alter its functionality [17,18]. Different studies suggest that the A allele is associated with lower gene expression, resulting in a decrease in aerobic capacity and metabolic efficiency during prolonged exercise [19,20]. On the other hand, the G allele has been linked to higher expression of *PPARGC1A* and better adaptation to endurance training, potentially conferring an advantage for individuals with this genetic predisposition in endurance sports [21,22].

To date, extensive research has supported the relationship between the *PPARGC1A* gene and physical performance in both endurance and strength sports. This gene plays a crucial role in muscle adaptation to exercise, promoting mitochondrial biogenesis and fatty acid oxidation, key elements for aerobic performance. In particular, the variant c.1444G>A is associated with the conversion of type II (fast) muscle fibers to type I (slow) fibers, which are more fatigue resistant, a valuable aspect in endurance sports such as cycling [23,24,25].

The relationship between the *PPARGC1A* gene and physical performance also encompasses strength and power sports. Some studies indicate that the activation of *PPARGC1A* not only improves muscle endurance but also influences the development of muscle strength and power [15,26,27]. This occurs through its impact on fatty acid oxidation and modulation of muscle fiber type, particularly in the transformation of type II muscle fibers into type I fibers, which are more fatigue resistant. These differences in fiber composition indicate that *PPARGC1A* influences not only endurance but also the modulation of muscle power and strength, especially relevant in sports that require a balance between aerobic and anaerobic endurance [28].

Therefore, the aim of this narrative review was to analyze the role of the *PPARGC1A* rs8192678 polymorphism in influencing metabolic efficiency and muscle fiber composition, with implications for both aerobic endurance and anaerobic performance in athletes, investigate the association between *PPARGC1A* gene variants and susceptibility to metabolic disorders, exploring their potential impact on energy homeostasis and exercise adaptation mechanisms, and assess the contribution of the rs8192678 polymorphism to mitochondrial biogenesis, fatty acid oxidation, and carbohydrate metabolism, and its relevance to optimizing athletic performance in endurance and strength sports.

## 2. Methods

### 2.1. Study Design

This narrative review specifically explores the role of the *PPARGC1A* gene, particularly its rs8192678 polymorphism, on sport-related phenotypes such as aerobic capacity, muscle adaptation, and sports performance. The association with metabolic diseases, such as type 2 diabetes and obesity, is addressed to provide additional context but is not the primary focus of this review. To ensure clarity, the author separated discussions on sports-related modalities and disease implications in the influence on aerobic capacity in skeletal muscle, relevance in power sports and muscle adaptation.

### 2.2. Search Strategy

A systematic literature search was conducted in accordance with PRISMA (Preferred Reporting Items for Systematic Reviews and Meta-Analyses) guidelines [29]. The following databases were used: PubMed, Scopus, Science Direct, and Web of Science—for their interception to October 2024.

The search terms were selected to include keywords and Medical Subject Headings (MeSH) related to the *PPARGC1A* gene and its rs8192678 polymorphism. The primary focus was on sports-related performance metrics. The search terms included combinations of:

(((“*PPARGC1A*” OR “rs8192678” OR “Gly482Ser”))) AND ((“aerobic capacity” OR “endurance” OR “muscle adaptation” OR “sports performance”)) AND (“metabolic diseases” OR “athletes”).

Inclusion criteria were (i) studies investigating the role of *PPARGC1A* rs8192678 in physical performance, aerobic capacity, muscle adaptation, and related traits in athletes or physically active populations, (ii) metabolic diseases related to these physical performance traits, and (iii) experimental, observational, and review articles were considered. Exclusion criteria were (i) articles focusing solely on metabolic diseases without addressing sports-related outcomes, (ii) studies on non-human subjects, and (iii) publications without available full text in English or Spanish.

### 2.3. Data Extraction and Analysis

Screened articles were based on the title and abstract for relevance to sports performance. Full-text screening was performed for potentially eligible studies, with disagreements resolved by consensus. Data on study design, population characteristics, and outcomes related to the rs8192678 polymorphism and its effects on performance metrics were extracted. Findings were synthesized qualitatively to assess the influence of genetic, environmental, and training factors on sports phenotypes.

The review acknowledges potential publication bias and variability in study designs, including differences in population genetics and training methodologies. A narrative synthesis was chosen due to heterogeneity across studies.

## 3. Influence on Aerobic Capacity in Skeletal Muscle

Aerobic capacity, which measures the efficiency with which the body uses oxygen during prolonged exercise, is closely related to the quantity of active mitochondria in skeletal muscle. In this sense, PGC-1α is a key regulator in the creation of new mitochondria (mitochondrial biogenesis), which increases the muscle’s capacity to generate energy through oxidative phosphorylation during aerobic exercise [17,30].

### 3.1. Association of the G Allele of the rs8192678 Polymorphism

The G allele of the rs8192678 polymorphism in the *PPARGC1A* gene has been associated with advantages in mitochondrial efficiency, particularly in aerobic capacity. This favors an increase in mitochondrial quantity and optimizes energy reserve utilization in endurance sports [31]. Several studies suggest that the presence of the G allele may be linked to aerobic adaptations in endurance athletes, such as long-distance runners and cyclists, enhancing their response to prolonged training by increasing lipid oxidation and the proportion of type I muscle fibers, which are more efficient for endurance performance [32,33,34,35,36].

This polymorphism appears to be associated with a higher proportion of oxidative muscle fibers in individuals carrying the G allele, enabling more efficient energy utilization during prolonged exercise and potentially explaining the genetic predisposition of some athletes to excel in endurance sports [37]. Recently, Hall et al. demonstrated that the G allele in the *PPARGC1A* gene is linked to efficiency in endurance activities, likely due to enhanced mitochondrial activity. Their findings indicate that carriers of this allele may have an advantage in endurance sports by optimizing energy metabolism in muscle cells [21]. In the study by Yvert et al., variability in muscle fiber composition in women was investigated, focusing on the proportion of type I and type II fibers, which influence endurance and explosive strength, respectively. These findings suggest that the distribution of these fibers in women could be genetically influenced, affecting their performance in various physical activities [25]. Furthermore, the study conducted by Lucia et al. found that elite cyclists carrying the G allele exhibited greater endurance capacity and faster post-exercise recovery compared to carriers of the A allele [38]. These studies have direct implications for personalized training, as genetic predisposition can be leveraged to maximize performance in endurance sports based on muscle composition.

### 3.2. Impact of the A Allele of the rs8192678 Polymorphism

Conversely, the A allele of the rs8192678 polymorphism in the *PPARGC1A* gene has been associated with reduced aerobic capacity and metabolic efficiency, potentially negatively impacting endurance sports performance. Several studies indicate that carriers of the A allele exhibit lower aerobic capacity and decreased metabolic efficiency in oxygen utilization during exercise. This diminished capacity can lead to reduced performance, particularly in endurance-intensive activities [32,38]. The presence of the A allele has also been linked to decreased mitochondrial biogenesis, meaning the muscle cells of these individuals are less efficient at producing and utilizing energy [39,40,41]. Other studies have demonstrated an association between the A allele and reduced oxidative capacity, which directly impacts performance in activities such as cycling, marathon running, and other long-duration sports [32,38,42]. This limited capacity for efficient energy generation results in a greater tendency for muscle fatigue in these athletes, affecting both the duration of physical effort and recovery between training sessions. Reduced mitochondrial biogenesis implies poorer adaptability to aerobic training and oxygen utilization. Consequently, individuals carrying the A allele may experience a less favorable response to aerobic training, leading to smaller improvements in endurance and suboptimal adaptation compared to athletes without this allele [43]. In studies involving long-distance runners and cyclists, it has been observed that A allele carriers may have a reduced ability to sustain prolonged efforts and exhibit greater lactate accumulation, which decreases exercise efficiency [44,45].

## 4. Relevance in Power Sports

While most studies have assessed the impact of the rs8192678 polymorphism in the *PPARGC1A* gene within endurance sports, some have also explored its role in power-based disciplines [15,46]. In these contexts, the influence of this polymorphism appears less defined. Although the G allele seems to confer an aerobic advantage, power sports are less reliant on oxidative metabolism, resulting in a less pronounced performance difference between the two alleles. However, some studies have suggested that the activation of metabolic pathways regulated by the PGC-1α protein may also support muscle recovery in strength sports, potentially benefiting athletes in disciplines requiring repeated anaerobic efforts [47,48].

A recent review conducted by Konopka et al. on studies investigating the rs8192678 polymorphism suggests that the G allele is associated with greater efficiency in endurance sports, likely due to enhanced aerobic and metabolic adaptations [49]. However, the effects of the A allele are not universally negative and may vary depending on the specific sports discipline and interactions with other genetic and environmental factors. Identifying these polymorphisms could be valuable not only for talent selection in sports but also for optimizing individualized training protocols to enhance performance.

## 5. Muscle Adaptation

The *PPARGC1A* gene regulates several key processes in muscle adaptation, particularly in response to endurance training [50]. Its activation leads to the formation of new mitochondria in muscle fibers, increasing the capacity of these cells to generate energy through oxidative phosphorylation [50,51,52]. This process is essential in endurance sports, where high efficiency in aerobic energy production is vital for performance.

In addition to mitochondrial biogenesis, PGC-1α promotes the transition of type II muscle fibers (fast-twitch fibers) into type I fibers (slow-twitch fibers), enhancing the muscle’s endurance capacity. Type I fibers are more efficient in oxygen utilization and fatty acid metabolism, enabling greater muscular endurance during prolonged periods of aerobic exercise [53,54].

### 5.1. Impact of the rs8192678 Polymorphism on PGC-1α Expression and Sports Performance

PGC-1α is a key regulator of mitochondrial biogenesis and oxidative phosphorylation. The rs8192678 polymorphism in the *PPARGC1A* gene has been shown to increase PGC-1α protein expression. The GG genotype of the *PPARGC1A* gene has been associated with increased PGC1α expression and enhanced fat oxidation capacity, whereas the Ser allele is linked to reduced efficiency in these processes. The elevated PGC1α expression in G carriers may result from greater mRNA stability, promoting more efficient protein synthesis, and improved interaction of PGC1α with transcription factors such as NRF1 and ERRα, which enhances the activation of genes involved in mitochondrial biogenesis [55]. Future research should focus on exploring the functional implications of the rs8192678 polymorphism across diverse populations and its impact on exercise performance and metabolic health. Additionally, investigating the potential therapeutic applications of modulating PGC-1α expression could provide insights into interventions for metabolic disorders and improve athletic performance. For instance, individuals carrying the A allele exhibit impaired exercise-induced transformation of muscle fibers from fast-twitch to slow-twitch, which are more oxidative and fatigue resistant [56]. The G allele is associated with enhanced activation of PGC-1α, leading to greater mitochondrial biogenesis capacity and fatty acid oxidation, which are essential for muscular energy efficiency [57]. This allele has been linked to improved performance in endurance sports, as muscle fibers in carriers of the G allele in the *PPARGC1A* gene tend to be more efficient in oxygen utilization and aerobic energy production. Additionally, athletes carrying the G allele have demonstrated a superior response to endurance training, showing improvements in aerobic capacity and prolonged endurance [32,38,43]. In contrast, the A allele has been associated with reduced PGC-1α expression, resulting in a diminished capacity for muscular adaptation to exercise [58].

In a study involving 197 sporadic amyotrophic lateral sclerosis (ALS) patients and 197 healthy controls, the rs8192678 polymorphism was examined for its role in oxidative stress responses during physical exercise. The study found that ALS patients with the AA genotype exhibited significantly higher lactate levels and greater protein oxidative products during exercise compared to those with the GG or GA genotypes. This suggests that the A allele may be associated with increased exercise-related oxidative stress [59]. A meta-analysis by Chen et al. examining athletes identified the A allele as being associated with reduced recovery capacity and impaired mitochondrial biogenesis efficiency. These characteristics may contribute to diminished performance in prolonged endurance activities and increased susceptibility to muscle fatigue [46]. However, studies exploring the relationship between the rs8192678 polymorphism and athletic performance have produced inconsistent findings. In certain cases, carriers of the G allele have exhibited significant advantages in endurance sports such as cycling, marathon running, and triathlon [21]. Conversely, other studies have reported no significant associations, suggesting that the effects of this polymorphism may be influenced by interactions with other genetic, environmental, and training-related factors [35,36,37]. Notably, one study on untrained men undergoing a 10-week endurance training program revealed that those with the rs8192678 polymorphism did not show the same increase in slow-twitch muscle fibers as those without the polymorphism. This indicates that the rs8192678 polymorphism may impair the adaptation of muscle fibers to endurance training, potentially affecting aerobic capacity and endurance performance [56].

Nonetheless, further research is required to better understand the interactions between this polymorphism, other genes, and external factors influencing muscle adaptation and athletic performance. To date, the most relevant studies associated with the rs8192678 polymorphism of the *PPARGC1A* gene are those presented in Table 1.

Chronic endurance exercise induces multiple adaptations in skeletal muscles, many of which are regulated by the expression of the PGC-1α protein. These adaptations are critical for performance in sports such as cycling, long-distance running, triathlon, and others [37,64,65].

### 5.2. Muscle Adaptations Induced by the PGC-1α Protein

The upregulation of PGC-1α expression promotes the transformation of type II muscle fibers (fast-twitch, less fatigue resistant) into type I fibers (slow-twitch, highly fatigue resistant). Type I fibers exhibit greater mitochondrial density and are more efficient at utilizing oxygen to generate energy, making them ideal for prolonged endurance activities [66,67,68]. PGC-1α induces the expression of enzymes involved in oxidative phosphorylation and the Krebs cycle, thereby enhancing the muscle’s ability to metabolize fats and carbohydrates—an essential factor for performance in prolonged endurance sports [69].

PGC-1α also stimulates the expression of glucose transporters such as GLUT4, facilitating greater glucose uptake by the muscle during exercise, a critical energy source for long-duration endurance events [70,71]. By improving oxidative capacity and energy metabolism, increased mitochondrial activity helps reduce the production of reactive oxygen species (ROS), which are harmful to muscle cells. This contributes to better recovery after intense exercise and reduces fatigue [72,73].

Although PGC-1α is extensively studied for its role in endurance sports [74], recent research has begun to investigate its influence on strength and power sports [57,75]. While its role in these disciplines is not as prominent as in aerobic activities, key mechanisms associated with PGC-1α may still contribute to performance improvements in sports requiring muscle power and explosiveness.

While PGC-1α promotes the transformation of type II (fast-twitch) muscle fibers into type I (slow and more oxidative) fibers, evidence suggests it can also enhance the metabolic efficiency of type II fibers without altering their typology. This is particularly important in strength and power sports, such as weightlifting or sprinting, where fast-twitch fibers are critical [56,76,77]. These findings support the role of PGC-1α in enhancing the metabolic efficiency of type II muscle fibers, which is vital for sports that demand strength and power.

Oxidative stress is a limiting factor in high-intensity strength sports. PGC-1α not only regulates mitochondrial biogenesis but also enhances the antioxidant capacity of skeletal muscle. This allows athletes to recover more quickly after intense training sessions or competitions, which is critical in sports that require multiple repetitions or explosive efforts within a short period. Notably, an increase in nuclear PGC-1α protein levels and mitochondrial gene expression has been observed three hours post-exercise, followed by an increase in mitochondrial protein content and enzymatic activity after 24 h [78]. In this context, the study by Tadaishi et al. demonstrated that PGC-1α increases the expression of antioxidant enzymes, such as SOD2 (superoxide dismutase 2), which protect muscle cells from free radical damage generated during intense exercise [10].

Differences in PGC-1α expression due to genetic variations, such as the rs8192678 polymorphism, provide insights into how genes interact with training to influence athletic performance. While the G allele appears to be associated with greater aerobic capacity, the effects of the A allele are not necessarily negative; instead, they may be more aligned with strength-based sports or those requiring a balance between endurance and power. However, further studies are needed to confirm the findings obtained so far.

### 5.3. Association Between Metabolic Diseases, Endurance/Strength Phenotypes, and the Potential Role of rs8192678

The rs8192678 polymorphism of the *PPARGC1A* gene, beyond its role in sports performance, has a significant impact on metabolism regulation, and its association with various metabolic diseases has been extensively studied.

#### 5.3.1. Type 2 Diabetes (T2DM)

The rs8192678 polymorphism, specifically the A allele, has been associated with reduced insulin sensitivity, a key factor in T2DM pathogenesis. PGC-1α regulates genes involved in glucose uptake and mitochondrial function, processes vital for efficient energy metabolism. Carriers of the A allele demonstrate diminished PGC-1α activity, impairing glucose oxidation and mitochondrial biogenesis. This results in higher fasting glucose levels and reduced glucose utilization during both rest and exercise [79,80].

A study involving Chinese adults found that non-diabetic subjects with the AA genotype had significantly higher fasting insulin levels and homeostasis model assessment-estimated insulin resistance (HOMA-IR) indices, indicating reduced insulin sensitivity [81]. Subjects of the AA genotype exhibit lower expression of genes involved in glucose oxidation and mitochondrial biogenesis, leading to poorer glucose utilization and higher fasting blood glucose levels. In a study of the northern Chinese population, logistic regression analysis showed that the AA genotype was associated with a 1.645-fold higher risk of T2DM, suggesting impaired glucose uptake [82]. These metabolic deficiencies have downstream effects on endurance and strength phenotypes, as glucose availability is critical for sustained aerobic and anaerobic performance. Furthermore, the presence of the A allele exacerbates glucose intolerance in conjunction with risk factors such as obesity and physical inactivity, further impairing physical performance [83,84,85].

#### 5.3.2. Obesity and Metabolic Syndrome

PGC-1α regulates fatty acid oxidation in mitochondria, a process that is critical for fat burning. The A allele is associated with reduced PGC-1α activity, leading to decreased lipid oxidation and increased fat storage. According to a meta-analysis, the c.1444G<A polymorphism impacts components of metabolic syndrome, including lipid metabolism, particularly in Asian populations [86].

The A allele may impair the body’s ability to generate heat from brown fat, thus reducing resting energy expenditure. This is supported by evidence showing that individuals with the A allele have impaired exercise-induced muscle fiber transformation, which is linked to thermogenic capacity [56].

Higher levels of inflammatory markers, such as C-reactive protein (CRP), have been observed in carriers of the A allele. A study on ALS patients found that those with the A allele exhibited increased oxidative stress and inflammation during exercise [59]. This suggests a potential link between the A allele and heightened inflammatory responses.

The A allele has been linked to higher body mass index (BMI) and waist circumference, indicators of central obesity. A meta-analysis involving 13,949 individuals found that the A allele is associated with higher blood pressure, a component of metabolic syndrome, particularly in younger adults [87]. In a study involving postmenopausal women, carriers of the A allele had a 25% increased risk of developing metabolic syndrome compared to G carriers [83].

Notably, dietary and exercise interventions, including resistance training and low-fat diets, have been shown to differentially affect lipid and glucose metabolism based on *PPARGC1A* genotypes. Tailored interventions targeting fat oxidation and mitochondrial function could mitigate the negative metabolic and performance outcomes in A allele carriers [88].

#### 5.3.3. Dyslipidemia and Cardiovascular Diseases

The rs8192678 polymorphism of the *PPARGC1A* gene is associated with dyslipidemia and cardiovascular diseases, as evidenced by several studies. The study of Jemaa et al. found a significant association between the rs8192678 polymorphism and T2DM, showing T2DM patients a higher frequency of the A allele compared to controls [84]. Indeed, the study of Zhang et al. in a Chinese population, the GA genotype of this polymorphism displayed a higher frequency in coronary artery disease (CAD) patients than in control subjects, indicating an increased risk of CAD associated with this genotype [89].

An intervention study carried out by Tobina et al. found that the GG genotype influences the effects of moderate-intensity exercise training on low-density lipoprotein cholesterol and total cholesterol concentrations [88]. The AA genotype was also associated with higher concentrations of LDL cholesterol in diabetic subjects, contributing to cardiovascular risk in T2DM [90].

From a performance perspective, these lipid imbalances and associated inflammatory states can compromise endurance and strength phenotypes, as efficient lipid metabolism and cardiovascular health are essential for sustained physical exertion. The interplay between metabolic diseases and physical performance underscores the importance of genetic screening in designing personalized exercise and nutrition programs to optimize both health and athletic outcomes.

## 6. Conclusions

The rs8192678 polymorphism of the *PPARGC1A* gene significantly influences both sports performance and metabolic health. The G allele is linked to improved mitochondrial efficiency, aerobic capacity, and endurance through enhanced oxidative metabolism and type I muscle fiber adaptation, making it advantageous for endurance sports. Conversely, the A allele impairs mitochondrial biogenesis and glucose utilization, increasing susceptibility to type 2 diabetes and obesity, but may offer potential benefits in strength-based sports. These findings highlight the importance of integrating genetic screening and personalized interventions in optimizing athletic performance and metabolic risk management.

Athletes with the G allele may benefit from endurance-focused training programs that optimize lipid utilization, while A allele carriers might prioritize strategies combining strength and endurance with an emphasis on enhancing mitochondrial biogenesis. Personalized dietary interventions are also relevant, with A allele carriers requiring strategies to improve insulin sensitivity and lipid metabolism and G allele carriers benefiting from diets rich in complex carbohydrates and healthy fats to maximize oxidative capacity. Early genotyping of rs8192678 could support talent identification, tailored training, and dietary plans, as well as aid in preventing metabolic diseases like T2DM by implementing targeted interventions in at-risk populations.

## Figures and Tables

**Table 1:** rs8192678 polymorphism of the *PPARGC1A* gene and its association with athletic performance.

Author (Year)	Population/Sample	Sport/Discipline	Design/Methodology	Focus	Results	Conclusion
Lucia et al. (2005) [38]	104 elite Spanish cyclists	Cycling	Case-control study	Endurance	The G allele was more prevalent in elite cyclists.	Possible association of the G allele with better aerobic capacity.
Ahmetov et al. (2009) [35]	450 Russian athletes (various sports)	Various (strength and endurance)	Case-control study	Endurance	The G allele was more frequent in endurance sports.	The polymorphism may influence aerobic performance.
Eynon et al. (2010) [60]	155 Israeli athletes	Athletics (long and middle distance)	Case-control study	Endurance and Strength	The A allele showed no significant differences in runners.	No direct association with athletic performance was found.
He et al. (2008) [61]	315 Chinese participants	Various (strength and endurance)	Cohort study	Endurance and Strength	Increased frequency of the G allele in endurance athletes.	The G allele may be associated with improved endurance capacity.
Ahmetov et al. (2007) [57]	700 Russian athletes	Strength and endurance	Case-control study	Endurance and Strength	Higher prevalence of the A allele in strength athletes.	The A allele may be linked to muscle strength.
Ruiz et al. (2009) [62]	106 elite Spanish rowers	Rowing	Case-control study	Strength	No significant difference observed between rowers and controls.	The polymorphism does not appear to directly influence rowing performance.
Ben-Zaken et al. (2015) [63]	120 elite Israeli athletes	Cycling and triathlon	Case-control study	Endurance	Higher frequency of the G allele in elite triathletes.	The G allele may be associated with aerobic endurance in combined sports.
Wang et al. (2016) [51]	240 Chinese athletes	Athletics (middle distance)	Cohort study	Strength	The G allele was associated with higher VO_2_max in runners.	The G allele appears to enhance aerobic capacity in long-distance runners.

## Data Availability

No new data were created or analyzed in this study. Data sharing is not applicable to this article.
